# Rationale and study protocol for the supporting children’s outcomes using rewards, exercise and skills (SCORES) group randomized controlled trial: A physical activity and fundamental movement skills intervention for primary schools in low-income communities

**DOI:** 10.1186/1471-2458-12-427

**Published:** 2012-06-12

**Authors:** David R Lubans, Philip J Morgan, Kristen Weaver, Robin Callister, Deborah L Dewar, Sarah A Costigan, Tara L Finn, Jordan Smith, Lee Upton, Ronald C Plotnikoff

**Affiliations:** 1 School of Education, Priority Research Centre in Physical Activity and Nutrition, University of Newcastle, Newcastle, Callaghan Campus, Australia; 2 School of Health Sciences, Priority Research Centre in Physical Activity and Nutrition, University of Newcastle, Newcastle, Callaghan Campus, Australia

## Abstract

**Background:**

Many Australian children are insufficiently active to accrue health benefits and physical activity (PA) levels are consistently lower among youth of low socio-economic position. PA levels decline dramatically during adolescence and evidence suggests that competency in a range of fundamental movement skills (FMS) may serve as a protective factor against this trend.

**Methods/design:**

The Supporting Children’s Outcomes Using Rewards Exercise and Skills (SCORES) intervention is a multi-component PA and FMS intervention for primary schools in low-income communities, which will be evaluated using a group randomized controlled trial. The socio-ecological model provided a framework for the 12-month intervention, which includes the following components: teacher professional learning, student leadership workshops (including leadership accreditation and rewards, e.g., stickers, water bottles), PA policy review, PA equipment packs, parental engagement via newsletters, FMS homework and a parent evening, and community partnerships with local sporting organizations. Outcomes will be assessed at baseline, 6- and 12-months. The primary outcomes are PA (accelerometers), FMS (Test of Gross Motor Development II) and cardiorespiratory fitness (multi-stage fitness test). Secondary outcomes include body mass index [using weight (kg)/height (m^2^)], perceived competence, physical self-esteem, and resilience. Individual and environmental mediators of behavior change (e.g. social support and enjoyment) will also be assessed. The System for Observing Fitness Instruction Time will be used to assess the impact of the intervention on PA within physical education lessons. Statistical analyses will follow intention-to-treat principles and hypothesized mediators of PA behavior change will be explored.

**Discussion:**

SCORES is an innovative primary school-based PA and FMS intervention designed to support students attending schools in low-income communities to be more skilled and active. The findings from the study may be used to guide teacher pre-service education, professional learning and school policy in primary schools.

**Trial registration:**

Australian New Zealand Clinical Trials Registry No: ACTRN12611001080910

## Background

Participation in physical activity (PA) is essential for optimizing children’s physical, social, cognitive and psychological development [1,2]. Activity of vigorous intensity may have additional benefits for young people, as physical fitness is a better predictor of metabolic health than total PA [3-5]. Unfortunately lack of PA among children and adolescents is a global concern [6] and current estimates suggest that only 50% of Australian primary school-aged children are meeting the current PA guidelines (i.e., 60 minutes/day of moderate-to-vigorous PA) [7]. In particular, promoting PA among youth from disadvantaged backgrounds is a public health priority because these individuals have reduced access to PA facilities and resources [8,9] and are often less active than those of middle and high socio-economic position [10-12].

The school setting is an ideal environment for the promotion of PA among youth as schools have the necessary equipment, personnel, facilities and curriculum to promote and provide opportunities for PA [13,14]. Numerous school-based PA interventions have been evaluated [15,16], including those specifically targeting youth from low-income backgrounds [17,18]. Multi-component school-based interventions that involve parents and encourage PA within and beyond the school day, are more efficacious than curriculum only interventions [15,16]. Although the evidence for effective school-based interventions is strong, studies rarely report their effect on movement skill competency. This is a notable omission because PA levels decline dramatically during adolescence [19,20] and evidence suggests that failure to attain competency may contribute to this decline, whereas competency may serve as a protective factor against this trend [21,22].

Proficiency in a range of fundamental movement skills (FMS) is considered to be the foundation for an active lifestyle [23] and the primary school years represent the “golden years” of motor skill development [23,24]. FMS include locomotor (e.g., running and hopping), object control (e.g., catching and throwing) and stability (e.g., balancing and twisting) skills [23]. These skills are ideally developed in childhood and subsequently refined into context- and sport-specific skills [24-26]. A recent systematic review of the health benefits associated with FMS competency found strong evidence for a positive association between FMS competency and PA in children and an inverse relationship between skill level and weight status [27]. Teaching movement skills improves both actual and perceived competence [28,29], both of which are important for future PA [30,31]. Indeed, lack of confidence in the physical domain is a major barrier to PA among many children and adolescents [32-34]. Alarmingly, many children finish primary school without achieving mastery in a range of FMS and those from disadvantaged backgrounds often demonstrate the lowest competency levels [11,35].

The low PA and poor FMS competency observed among children living in low-income communities can be explained by socio-environmental factors (e.g., working parents, lack of PA opportunities and unsafe neighborhoods etc.) [36,37], but may also reflect a failure of current school-based programs and strategies [38]. Indeed, the recent Crawford report highlighted both the central role that schools play in the promotion of PA and the dire state of PE and school sport in Australian primary schools [38]. Formative research conducted by Morgan and colleagues indicated that the crowded school curriculum along with inadequate teacher training programs contributes to teachers’ reluctance to teach PE and the poor quality of existing PE programs [39,40]. Combined, these findings illustrate the importance of designing and evaluating school-based approaches to PA promotion among the most vulnerable individuals (i.e., those living in low-income communities). This paper provides the rationale and methods for the Supporting Children’s Outcomes Using Rewards Exercise and Skills (SCORES) intervention. SCORES is a multi-component school-based intervention that combines a range of evidence-based behavior change strategies to promote PA and FMS competency among primary school aged children from low-income communities.

## Methods/design

### Study design

**Figure 1:**
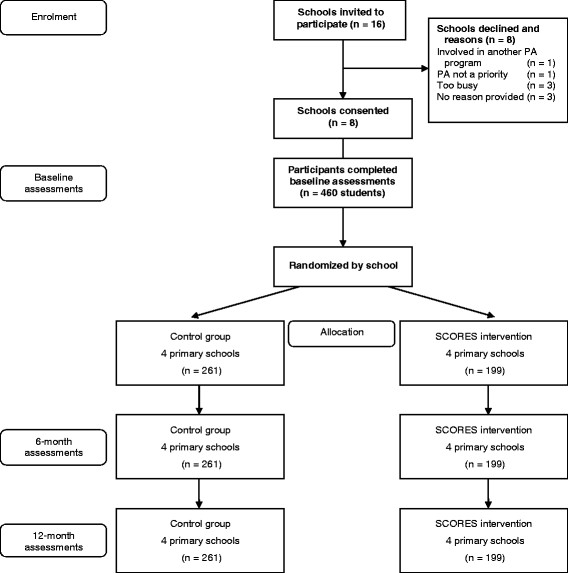
Study design and flow.

The SCORES intervention will be evaluated using a group randomized controlled trial (Figure 1). The 12-month multi-component PA and FMS intervention will target children in grades 3 and 4 (ages 7 to 10 grades) in eight primary schools. Assessments were conducted at baseline [February–March (Term 1) 2012], and will be repeated mid-program [August–September (Term 3) 2012] and at 12-months post baseline [February–March (Term 1) 2013]. The design, conduct and reporting of this group RCT will adhere to the Consolidated Standards of Reporting Trials (CONSORT) guidelines for group trials [41]. Ethics approval for this study was obtained from the Human Research Ethics Committees of the University of Newcastle, Australia and the New South Wales (NSW) Department of Education and Communities. School Principals, teachers, parents and study participants provided written informed consent. 

### Setting and participants

The Socio-Economic Indexes for Areas (SEIFA) index of relative socioeconomic disadvantage was used to identify eligible primary schools. The SEIFA index (scale 1 = *lowest* to 10 = *highest*) summarizes the characteristics of people and households within an area and was developed using the following data: employment, education, financial well-being, housing stress, overcrowding, home ownership, family support, family breakdown, family type, lack of wealth (no car or telephone), low income, Indigenous status and foreign birth. Sixteen government primary schools located within 30 minutes drive from the University of Newcastle, with a SEIFA index of ≤ 5 (lowest 50%) were invited to participate in the study and eight schools consented to participate (50% consent rate). All students in grades 3 and 4 (Stage 2) at the study schools were invited to participate in the program. From the 592 eligible children at the eight schools, 460 children consented to participate (78% consent rate).

### Sample size calculation

Power calculations were conducted to determine the sample size required to detect changes in the three primary outcomes [i.e., PA, cardiorespiratory fitness (CRF) and FMS] at the 12-month assessments. All calculations assumed baseline-posttest correlation scores of 0.80 and were based on 80% power with alpha levels set at *p* < 0.05. Using the standard deviation (SD = 33) and intraclass correlation coefficient (ICC = 0.05) values from the Kinder-Sportstudie (KISS) [42], it was calculated that a study sample of N = 440, with 8 clusters (i.e. schools) of 55 students would provide adequate power to detect an achievable between group difference of 11 moderate-to-vigorous physical activity (MVPA) minutes/day [42]. Based on data from the Action Schools BC! (SD = 13) [43] and the KISS (ICC = 0.03) [42] studies, a sample of 440 would also provide adequate power to detect a between group difference of 4 laps on the multi-stage fitness test (i.e., CFR outcome). In the absence of existing ICC values for FMS outcomes, an ICC estimate of 0.05 and a SD of 15 units [44] indicated that the study would be adequately powered to detect a between group difference of 5 units on the TGMD-II gross motor quotient.

### Blinding and randomization

Baseline assessments were conducted prior to randomization by trained research assistants. The intervention will be evaluated using a group RCT-design and schools were randomly allocated to the control or intervention groups for the duration of the study. Schools were match-paired based on their size and SEP (based on post-code of school) then randomly allocated to the intervention or control group using a computer-based random number producing algorithm by a researcher not involved in the current study. This method ensured that schools had an equal chance of allocation to each group.

### Intervention

**Figure 2:**
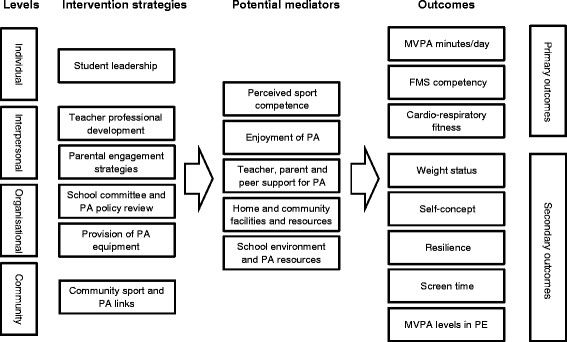
SCORES intervention components, potential mediators and outcomes.

SCORES is a 12-month multi-component PA and FMS intervention for primary schools in low-income communities (Figure 2). The socio-ecological model [45] provided a framework for the intervention components. Within this framework, behavior change strategies were guided by Self-Determination Theory (SDT) [46,47] and Competence Motivation Theory (CMT) [48,49]. SDT proposes that social-contextual factors (e.g., motivational strategies used by teachers and parents) can influence individuals’ motivation and subsequent behavior by satisfying three basic psychological needs: 1) *Autonomy*, the need to experience one’s behavior as self-endorsed or volitional; 2) *Competence,* the need to effectively interact with one’s environment and achieve positive outcomes; and 3) *Relatedness,* the need to feel supported and connected with others [46,47]. SDT has been used extensively with adolescents in PE-based research [50-53] and evidence suggests that students who feel self-determined are more engaged and more active in PE lessons [51,52]. In the context of PA promotion, CMT provides a theoretical link between FMS competence and PA [48]. While CMT includes competence and a construct similar to SDT’s relatedness (social support), it differs in its focus on enjoyment and includes global self-esteem as a predictor of behavior. Our integrated model proposes that children who have high levels of perceived and actual athletic competence, receive social support from significant others and feel a sense of control over their PA experiences will enjoy PA and seek opportunities to be active in the future. 

The SCORES intervention will be implemented in three phases. Phase 1 will focus on teacher professional learning, student workshops, provision of equipment and the establishment of a school committee. In Phase 2, the research team will work with the school committees to advocate for relevant policy change to promote PA and FMS. In addition, the research team will employ a range of strategies to engage parents and encourage them to support their children’s PA. Phase 3 will address strategies to improve school-community links (e.g., inviting local sporting organizations to assist with school sport programs). The focus of this phase will be program consolidation and the research team will work with schools to establish sustainability. The intervention components are detailed in Table 1 and a description of how the socio-ecological framed intervention will facilitate behavior change at the individual, interpersonal, organizational and community levels is provided below:

*Individual:* While the intervention will involve a number of indirect strategies to support and improve children’s PA behaviors (i.e., through teachers, parents and the community), students will be directly involved in the SCORES leadership workshops which will be delivered by the research team. The workshops will focus on developing leadership and organizational skills necessary for running lunch and recess PA sessions and assisting classroom teachers to deliver high quality PE lessons. These sessions will be designed to satisfy students’ basic psychological needs (i.e., autonomy, competence and relatedness). and enable them to achieve SCORES leadership accreditation.

*Interpersonal:* The SCORES intervention will target teachers, parents and the students themselves as facilitators of behavior change. The teacher professional learning workshops will provide opportunities for non-specialist PE (i.e., classroom teachers) to improve their teaching skills and knowledge in regards to PA promotion and FMS development. The workshops were guided by SDT and CMT and will be used to reinforce the SAAFE (Supportive, Active, Autonomous, Fair and Enjoyable) teaching principles, which were developed for the study and are described in Table 2.

**Table 1:** Intervention components, behavior change techniques and targeted constructs in the SCORES intervention

**Intervention component**	**Dose**	**Description**	**Behavior change strategies**	**Targeted constructs**
1) Student leadership	1 x 2 hours	Students will be provided with an opportunity to achieve SCORES leadership accreditation. Students will attend the SCORES leadership workshop, which will be delivered at the study schools during PE/sport by the research team. This will provide students with formal acknowledgment (i.e. certificates) and rewards (i.e. water bottles, stickers) for their participation. SCORES leaders will be encouraged to set up (i.e. equipment monitor), run (i.e. deliver lunch and recess activities) and promote (i.e. speak on assembly) PA and FMS development in the school setting. Students who complete these tasks will have the opportunity to achieve a yellow award (complete 5 tasks), red award (complete 10 tasks), and blue award (complete 15 tasks), and receive the associated rewards (i.e. certificates, wrist bands, hats, sporting equipment).	▪Provide instruction	▪Actual competence
			▪Model or demonstrate the behavior	▪Perceived competence
			▪Provide contingent rewards	▪Social support
			▪Prompt identification as a role model	▪Enjoyment
			▪Plan social support or social change	
			▪Set graded tasks	
2) Professional learning workshops for teachers	1 x full day for Stage 2 teachers^1^	The research team will deliver professional development workshops for teachers. Workshops will focus on effective teaching methods for the development of FMS, strategies for teaching and assessing FMS, increasing MVPA and enjoyment in PE and school sport (based on the SAAFE teaching principles).	▪Provide instruction	▪Social support
			▪Model or demonstrate the behavior	
			▪Time management	
			▪Provide feedback on performance	
	1 x half day for all teachers at intervention schools	*i) Stage 2 teachers’ workshop:* will be held at the university. This workshop will be provided for Stage 2 teachers only.		
		*ii) Whole-school workshops:* will be delivered in the study schools during one of their scheduled professional learning days.		
3) Parental engagement	4 x newsletters	*i) Newsletters -* Parents of study participants will be provided with newsletters to educate and encourage them to support their children’s PA behaviors. Newsletters will also provide updates and feedback on the project.	▪Provide information on consequences	▪Social support
			▪Provide feedback on performance	
			▪Plan social support or social change	
			▪Provide general encouragement	
	1 x Parent evening	*ii) FMS Homework* – Students will be encouraged to complete practical homework tasks focused on FMS development with their parents/guardians.		
	Weekly FMS homework	*iii) Parent evening –* Parents will be invited to attend an interactive information session on how to promote and increase PA and FMS in the home setting.		
4) Policy and environment	On-going	*i) School committee and policy review and recommendations:* The research team will conduct a review of PA policy in the schools. The research team will work with Principals and school committees to revise policy to support the PA promotion. Policy recommendations include:	▪Provide opportunities for behavior	▪Physical environment
			▪Provide access to equipment to encourage behavior	
		a) Functioning school PA committee (i.e., school committee to meet once a school term).		
		b) All students participate in at least 120 minutes of timetabled PA per week (i.e., ensure PE and school sport are timetabled).		
		c) 50% of PE and school sport time devoted to MVPA (i.e., lessons designed to maximize huff and puff activity).		
		d) Annual reporting of students’ FMS and fitness (e.g. report cards describing student levels).		
		e) Promotion of active playgrounds (e.g. organized activities and access to equipment).		
		f) Involve family members in school-based PA (e.g. parents as helpers in PE and school sport).		
		*ii) Equipment and resources:* Each school will be provided with PA equipment (e.g. bats, balls etc.) and resources (e.g. activity cards) to support the implementation of the intervention based on their individual school needs (approx. $1,000).		
5) Community links	6 x visits	Community organizations (e.g. local football clubs) will be invited to the visit the study schools during PE/school sport. This will help to promote community sporting links.	▪Provide instruction	▪Social support
			▪Model or demonstrate the behavior	
			▪Provide information about opportunities in the local environment	

*Note:* FMS = Fundamental Movement Skills; PE = physical education; SCORES = Supporting Children’s Outcomes using Rewards, Exercise and Skills.

^1^Stage 2 teachers are the classroom teachers of students in the intervention schools.

**Table 2:** SAAFE teaching principles and strategies

**Principles**	**Strategies**
**Supportive** – Lessons conducted in a supportive environment.	1. Publicly recognize all students’ effort, learning, achievements, and improvement.
	2. Provide feedback on student effort, process and progress (not results).
	3. Identify and manage inappropriate student behavior (e.g., teasing, over-competitiveness).
	4. Promote positive social interactions between students.
**Active** - Lessons involve a high level of movement and active learning time (ALT).	1. Use small-side games, circuits and tabloids to maximize participation.
	2. Ensure equipment is plentiful and developmentally appropriate.
	3. Monitor in-class physical activity using pedometers (i.e., approx. 75–85 steps/min of PE time is equal to 50% ALT).
	4. Use student leaders to set-up games and activities.
**Autonomous** – Lessons involve elements of choice and opportunities for graded tasks.	1. Ensure that tasks incorporate multiple challenge levels, and give students the freedom to select level of difficulty.
	2. Provide students with opportunities to create and modify rules and activities.
	3. Provide students with opportunities for leadership roles.
	4. Encourage students to assess their own skill performances (e.g., detect and correct their own errors).
**Fair** – Lessons provide all students with an opportunity to experience success.	1. Ensure tasks are not dominated by the most competent students.
	2. Modify the tasks to increase the opportunity for success (i.e., make the goals bigger, reduce the number of defensive players, alter the equipment used, revise the task rules).
	3. Ensure students are evenly matched in competitive activities.
	4. Acknowledge and reward participation and good sportsmanship.
**Enjoyable** – Lessons are designed to be enjoyable and engaging for all students.	1. Include a wide variety of games and activities.
	2. Provide engaging and age appropriate tasks.
	3. Avoid boring and repetitive activity (e.g., running around the field for a warm-up).
	4. Don’t use exercise or activity as punishment.

Parents will be engaged using the following strategies: a) newsletters describing intervention progress and encouraging PA and FMS practice, b) weekly FMS homework (using FMS activity cards to be completed by children at home under parental supervision), and c) a parent information evening focusing on parental strategies to promote PA and FMS development outside of school setting. Finally, students who have gained SCORES accreditation will be responsible for organizing recess and lunch-time physical activities for other students in the study schools.

*Organizational:* The research team will work with the schools to implement evidence-based policy and practice that is supportive of all students’ PA. The specific PA policies are provided in Table 1. A school committee will be established to guide a review of existing school policy and the implementation of new policies. The research team conducted an audit of each school’s equipment and resources. Intervention schools will be provided with PA equipment (e.g. bats, balls etc.) to support PA promotion, based on their individual requirements.

*Community:* The research team will conduct an audit of sport and PA organizations within each school’s local community. Community organizations will then be invited to visit schools during PE and school sport. The aim of this intervention component is to create partnerships between schools and community organizations. It will also serve to increase students’ awareness of, and participation in, extra-curricular sport and PA in their local community.

*Control group:* To prevent potential compensatory rivalry and resentful demoralization [54], the control schools will be provided with a condensed version of the program following the 12-month assessments. The condensed version of the program will include the professional learning workshops for teachers and students, strategies to engage parents and a review of school PA policy will be conducted. A PA equipment pack valued at approximately $1000 AUD (including pedometers, bats, balls, cones, goals etc.) will also be provided based on individual school requirements.

### Outcomes

Baseline assessments were conducted by trained research assistants at the study schools. Mid-intervention (6-months) and post-intervention (12-months) assessments will also be conducted at the study schools. For consistency and accuracy, a protocol manual, which includes specific instructions for conducting all assessments, was developed and will be used by research assistants. Questionnaires were completed before the physical assessments in exam-like conditions and physical assessments were conducted in a sensitive manner (e.g., weight measured in a discreet, private setting). Demographic information including age, gender, ethnicity, language spoken at home and mother/father’s highest level of school was collected at baseline. A range of primary and secondary outcomes and hypothesized mechanisms of behavior change will be measured.

### Primary outcomes

#### Physical activity

PA will be assessed using triaxial Actigraph accelerometers (GT3X and GT3X+), which will be worn by participants during waking hours for seven consecutive days, except while bathing and swimming. Trained research assistants, following standardized accelerometer protocols [55], will fit the monitors and explain the monitoring procedures to students. Data will be collected and stored in 10-second intervals. The mean activity counts per minute (CPM) and daily step counts will be calculated, thresholds for activity counts will be used to categorize PA into sedentary, light, moderate and vigorous intensity activity [56].

#### Cardio-respiratory fitness

Cardio-respiratory fitness (CRF) will be assessed using a 20 m multistage fitness test [57]. Participants will be required to run back and forth between two lines over a 20 m distance within a set time limit. Running speed will start at 8.5 km/hour and will increase by 0.5 km/hr each minute using the Multi-stage test cadence CD. Participants will be instructed to run in a straight line and to place one foot over the 20 m line before the next beep. The test is completed when a participant fails to reach the line for two consecutive shuttles. Scores will recorded as the level and shuttle reached, which will be converted to the number of 20 m laps completed to provide a continuous variable for analysis.

#### Fundamental movement skill competency

FMS competency will be assessed using the Test of Gross Motor development (TGMD) II [44]. The TGMD II includes six locomotor (i.e., run, gallop, hop, leap, horizontal jump, slide) and six object control (i.e., striking a stationary ball, stationary dribble, kick, catch, overhand throw, and underhand roll) skills. Participants will perform each skill twice and skills will be video-taped for assessment. Inter-rater and intra-rater reliability will be established (> 80%) using pre-coded video-tapes before movement skills are assessed.

### Secondary outcomes

#### Height and weight

Weight will be measured in light clothing without shoes using a portable digital scale (Model no. UC-321PC, A&D Company Ltd, Tokyo Japan) to the nearest 0.1 kg. Height will be recorded to the nearest 0.1 cm using a portable stadiometer (Model no. PE087, Mentone Educational Centre, Australia). Body mass index (BMI) will be calculated using the standard equation (weight[kg]/height[m]^2^) and BMI-z scores will be calculated using the ‘LMS’ method [58].

#### Self-concept

Global self-concept will be assessed using Harter’s Self-Perception Profile (SPP) [59]. The SPP utilizes a four-choice structured alternative format to reduce socially desirable responses. Participants must first decide which of the two statements best describes them and then choose whether the statement is ‘sort of true’ or ‘really true’ for them. Each item is scored from 1 (*low-self-perception*) to 4 (*high self-perception*).

#### Resilience

Participants will complete the Child and Youth Resilience Measure (CYRM-28) [60]. Based on a validation study involving children from 11 countries, the CYRM-28 was found to have good content-related validity and provide a culturally sensitive measure of youth resilience [61]. The CYRM-28 has 28 items and includes three sub-scales: individual, relationships with primary care-givers, and contextual factors that facilitate a sense of belonging. Items are rated on a 5-point Likert scale with values ranging from 1 (*Not at all*) to 5 (*A lot*).

#### Screen time

Participants will complete six items related to weekday and weekend day recreational screen time from the Health Behavior in School-aged Children (HBSC) study [62]. The HBSC screen time questions compare favorably with other measures in sedentary behavior [63] and has acceptable reliability in children, with intraclass correlation coefficients ranging from 0.86 (95% CIs, 0.76–0.92) for watching television on school days to 0.38 (95% CIs, 0.10–0.60) for using the internet for non-school purposes and chatting on line [64].

### Hypothesized mediators of behavior change

A poor understanding of the mechanisms of behavior change in PA interventions has been noted in the literature [65,66]. Students, parents and teachers will complete a range of scales assessing individual and socio-environmental level mediators of PA behavior change.

#### Perceived sport competence

Perceived sport competence will be assessed using a subscale from Harter’s SPP [59].

#### Enjoyment

Enjoyment of PA will be assessed using the Physical Activity Enjoyment Scale (PACES) [67]. The 16-item scale is scored on a 5-point Likert scales, with responses ranging from 1 (*Disagree a lot*) to 5 (*Agree a lot).*

#### Social support

Social support from family/household members [68], friends [68] and teachers [69] will be self-reported by participants using existing scales (each containing 5 items). All scales utilize 5-point Likert scales with responses ranging from 1 (*never*) to 5 (*always*). Parents will also report the level of social support they provide for their children using the Children’s Leisure Activities Study Survey (CLASS) [70].

#### Environment

Parents will complete selected scales from the CLASS assessing children’s access to PA facilities and equipment in their home and local community [70]. Parents will also report barriers and facilitators to their children’s PA in the local community using the CLASS. Teachers at the study schools will report on their schools’ physical environment and facilities and students’ access to these within and beyond the school day using scales selected from the New South Wales Schools Physical Activity and Nutrition Survey (SPANS) [71].

### Process evaluation

A range of process data will be collected to complement the outcome data. Process measures will include i) teacher and student attendance at workshops (i.e., percentage attendance), ii) student leadership accreditation (i.e., number of students who complete the workshop and satisfy the accreditation guidelines), iii) teacher satisfaction with professional learning workshops (using workshop evaluation questionnaires at the end of Phase 1), iv) parental involvement will be determined using a process evaluation questionnaire (e.g. reading newsletters and completion of home-based FMS tasks) and attendance at the parent evening, v) teacher, student and parent satisfaction with all intervention components (using process evaluation questionnaires at the completion of the study), vi) compliance with PA policies will be determined through interviews with school Principals, vii) PE intervention fidelity will be determined (using SOFIT observations). PE lessons will be observed at baseline, 6- and 12-months using the System for Observing Fitness Instruction Time (SOFIT) tool [72]. Percentage of lesson time spent in MVPA and time dedicated to skill development will be assessed. All teachers of Stage 2 students (both intervention and control groups) will be observed at each time point.

### Statistical methods

The study will be adequately powered to detect clinically important changes in the three primary outcomes at the 12-month assessments. Statistical analyses of the primary and secondary outcomes will be conducted using linear mixed models with PROC MIXED in SAS V 9.1 (SAS Institute Inc, Cary, NC) and alpha levels will be set at *p* < 0.05. The mixed models will be specified to adjust for the clustered nature of the data and will follow the intention to treat principle. Potential moderators of the intervention effects (e.g., ethnicity, socio-economic status and type of school) will be explored using linear mixed models. Differences between participants in the intervention and groups at baseline and differences between completers and those who drop out of the study will be examined using Chi square and independent samples t-tests in PASW Statistics 17 (SPSS Inc. Chicago, IL) software. Hypothesized mediators of PA behavior change will be examined using multilevel linear analysis and a product-of-coefficients test that is appropriate for cluster randomized controlled trials [73].

## Discussion

In this paper we described the rationale and study protocol for the SCORES intervention. To the authors’ knowledge, SCORES is the first PA and FMS intervention targeting Australian primary school children in low-income communities. Targeting children of low SEP is important because they have reduced access to PA opportunities and are typically less active and skilled than youth of middle and high SEP [11,12]. By Year 4, students should achieve mastery in a range of FMS, however, recent data suggests that the prevalence of advanced skills is low among Australian children and proficiency levels have declined since 2004 [11].

Although there is strong evidence that school-based PA interventions are effective in increasing the duration of PA and increasing CRF in children and adolescents, their impact on leisure time PA and FMS is less convincing [14-16]. Such programs are typically evaluated among youth transitioning from childhood to adolescence, a period of time that is characterized by an erosion of activity patterns [19,74]. Nevertheless, recent well-designed studies [42,43,75], such as the KISS intervention [42] have demonstrated that multi-component school-based interventions can increase PA and CRF in children. However, these studies have involved daily PE lessons, which may not be feasible in many schools.

Alternatively, interventions that provide professional learning opportunities for teachers and promote PA within existing PE lessons and throughout the school day (i.e., lunch time and recess) may provide a valuable framework for sustainable practice. Unfortunately, many primary school teachers lack the confidence and skills to teach PE effectively [40,76], which may explain their reluctance to teach this subject in favor of traditional academic subjects (e.g., mathematics and science). The lack of focus on teacher professional learning in school-based PA interventions is surprising considering the importance placed on professional learning in the general education literature [77] and that teachers have specifically stated that professional development in PE, and teaching FMS in particular, is urgently needed and a high priority for improving PA-related outcomes in primary schools [76].

SCORES is an innovative multi-component school-based intervention targeting primary school children in low-income communities. The strengths of this study include the study design, the objective measures of PA, FMS and CRF and the comprehensive multi-component intervention. The findings from the study may be used to guide teacher pre-service education, professional learning and school policy in primary schools.

## Abbreviations

CLASS: Children’s leisure activities study survey; CMT: Competence motivation theory; CYRM-28: Child and Youth Resilience Measure; HBSC: Health behavior in school-aged children; MVPA: Moderate-to-vigorous physical activity; PACES: Physical activity enjoyment scale; PA: Physical activity; Self-determination theory; SPP: Self-perception profile; SPANS: Schools physical activity and nutrition survey; System for observing fitness instruction time; TGMD-II: Test of gross motor development II.

## Competing interests

The authors have no competing interests to declare.

## Authors’ contributions

DRL, PJM, RC, and RCP obtained funding for the research. All authors contributed to developing the protocols and reviewing, editing, and approving the final version of the paper. DRL, PJM, RCP and KW developed the intervention materials. TF, JS, LU, SAC and DD are responsible for data collection and cleaning. DRL is the guarantor and accepts full responsibility for the conduct of the study and the integrity of the data. All authors have read and approved the final manuscript.

## Pre-publication history

The pre-publication history for this paper can be accessed here:

http://www.biomedcentral.com/1471-2458/12/427/prepub

## References

[B1] Strong WB Malina RM Blimkie CJ Daniels SR Dishman RK Gutin B Hergenroeder AC Must A Nixon PA Pivarnik JM Evidence based physical activity for school-age youth J Pediatr 2005 146 6 732 737 10.1016/j.jpeds.2005.01.055 15973308

[B2] Tomporowski PD Davis CL Miller PH Naglieri JA Exercise and children’s intelligence, cognition, and academic achievement Educ Psychol Rev 2008 20 2 111 131 10.1007/s10648-007-9057-0 19777141 PMC2748863

[B3] Ekelund U Poortvliet E Nilsson A Yngve A Holmberg A Sjostrom M Physical activity in relation to aerobic fitness in 14- to 15-year-old boys and girls Eur J Appl Physiol 2001 85 195 201 10.1007/s004210100460 11560070

[B4] Rizzo NS Ruiz JR Hurtig-Wennlof A Ortega FB Sjostrom M Relationship of physical activity, fitness, and fatness with clustered metabolic risk in children and adolescents: the European Youth Heart Study J Pediatr 2007 150 4 388 394 10.1016/j.jpeds.2006.12.039 17382116

[B5] Froberg K Anderson LB Mini Review: physical activity and fitness and its relations to cardiovascular disease risk factors in children Int J Obes 2005 29 S 34 39 10.1038/sj.ijo.0803096 16385750

[B6] Guthold R Cowan MJ Autenrieth CS Kann L Riley LM Physical activity and sedentary behavior among schoolchildren: a 34-country comparison J Pediatr 2010 157 1 43 49 10.1016/j.jpeds.2010.01.019 20304415

[B7] Hardy LL King L Espinel P Cosgrove C Bauman A NSW Schools Physical Activity and Nutrition Survey (SPANS) 2010: Full Report 2010 Sydney: NSW Ministry of Health

[B8] Gordon-Larsen P Nelson MC Page P Popkin BM Inequality in the built environment underlies key health disparities in physical activity and obesity Pediatr 2006 117 2 417 424 10.1542/peds.2005-0058 16452361

[B9] Moore LV Diez Roux AV Evenson KR McGinn AP Brines SJ Availability of recreational resources in minority and low socioeconomic status areas Am J Prev Med 2008 34 1 16 22 10.1016/j.amepre.2007.09.021 18083446 PMC2254179

[B10] Department of Health & Ageing 2007 Australian National Children’s Nutrition and Physical Activity Survey - Main Findings 2008 Commonwealth of Australia: ACT 35 38

[B11] Hardy LL King L Espinel P Cosgrove C Bauman A NSW Schools Physical Activity and Nutrition Survey (SPANS) 2010: Short Report 2011 Sydney: NSW Ministry of Health 10.1016/j.jsams.2011.03.003 21454126

[B12] Cleland VJ Ball K Magnussen C Dwyer T Venn A Socioeconomic position and the tracking of physical activity and cardiorespiratory fitness from childhood to adulthood Am J Epidemiol 2009 170 9 1069 1077 10.1093/aje/kwp271 19767351

[B13] Centers for Disease Control & Prevention School health guidelines to promote healthy eating and physical activity MMWR Morb Mortal Wkly Rep 2011 60 5 1 76 21918496

[B14] Dobbins M De Corby K Robeson P Husson H Tirilis D School-based physical activity programs for promoting physical activity and fitness in children and adolescents aged 6–18 Coch Data Sys Rev 2009 1 CD007651 10.1002/14651858.CD007651 19160341

[B15] Van Sluijs EMF McMinn AN Griffin SJ Effectiveness of interventions to promote physical activity in children and adolescents: systematic review of controlled trials Br J Sports Med 2008 42 653 657 18685076

[B16] Salmon J Booth ML Phongsavan P Murphy N Timperio A Promoting physical activity participation among children and adolescents Epidemiol Rev 2007 29 144 159 10.1093/epirev/mxm010 17556765

[B17] Nemet D Geva D Eliakim A Health promotion intervention in low socioeconomic kindergarten children J Pediatr 2011 158 5 796 801 10.1016/j.jpeds.2010.10.040 21244862

[B18] Lubans DR Morgan PJ Aguiar E Callister R Randomized controlled trial of the Physical Activity Leaders (PALs) program for low-active adolescent boys from disadvantaged secondary schools Prev Med 2011 52 239 246 21276812 10.1016/j.ypmed.2011.01.009

[B19] Nader PR Bradley RH Houts RM McRitchie SL O’Brien M Moderate-to-vigorous physical activity from ages 9 to 15 years JAMA 2008 300 3 295 305 10.1001/jama.300.3.295 18632544

[B20] Janz KF Dawson JD Mahoney LT Tracking physical fitness and physical activity from childhood to adolescence: the muscatine study Meed Sci Sports Exerc 2000 32 7 1250 1257 10.1097/00005768-200007000-00011 10912890

[B21] Barnett LM van Beurden E Morgan PJ Brooks LO Beard JR Childhood motor skill proficiency as a predictor of adolescent physical activity J Adolesc Health 2009 44 3 252 259 10.1016/j.jadohealth.2008.07.004 19237111

[B22] Lopes VP Rodrigues LP Maia JA Malina RM Motor coordination as predictor of physical activity in childhood Scand J Med Sci Sports 2011 21 5 663 669 10.1111/j.1600-0838.2009.01027.x 21917017

[B23] Gallahue DL Ozmun JC Understanding motor development: Infants, children, adolescents, adults 2006 6 Boston: McGraw-Hill

[B24] Clark JE Metcalfe JS Clark JE, Humprehy JH The mountain of motor development Motor development: Research and reviews. Volume 2 2002 Reston, VA: National Association of Sport & Physical Education 163 190

[B25] Stodden D Goodway JD Langendorfer S Roberton MA Rudisill ME Garcia C Garcia LE A developmental perspective on the role of motor skill competence in physical activity: an emergent relationship Quest 2008 60 290 306 10.1080/00336297.2008.10483582

[B26] Clark JE From the beginning: a developmental perspective on movement and mobility Quest 2005 57 37 45 10.1080/00336297.2005.10491841

[B27] Lubans DR Morgan PJ Cliff DP Barnett LM Okely AD Fundamental movement skills in children and adolescents: review of associated health benefits Sports Med 2010 40 12 1019 1035 10.2165/11536850-000000000-00000 21058749

[B28] Robinson LE Goodway JD Instructional climates in preschool children who are at-risk. Part I: object-control skill development Res Q Exerc Sport 2009 80 3 533 542 10.5641/027013609X13088500159480 19791639

[B29] Robinson LE Rudisill ME Goodway JD Instructional climates in preschool children who are at-risk. Part II: perceived physical competence Res Q Exerc Sport 2009 80 3 543 551 10.5641/027013609X13088500159525 19791640

[B30] Ulrich BD Perceptions of physical competence, motor competence and participation in organized sport: Their interrelationships in young children Res Q Exerc Sport 1987 58 57 67

[B31] Barnett LM Morgan PJ van Beurden E Beard JR Perceived sports competence mediates the relationship between childhood motor skill proficiency and adolescent physical activity and fitness: a longitudinal assessment Int J Behav Nutr Phys Act 2008 5 40 10.1186/1479-5868-1185-1140 PMC2569960 18687148

[B32] Baker BL Davison KK I know I can: a longitudinal examination of precursors and outcomes of perceived athletic competence among adolescent girls J Phys Act Health 2011 8 2 192 199 21415446 10.1123/jpah.8.2.192 PMC5500253

[B33] Raudsepp L Liblik R Hannus A Children’s and adolescents’ physical self-perceptions as related to vigorous physical activity and physical fitness Pediatr Exerc Sci 2002 14 97 106

[B34] Dishman RK Hales DP Pfeiffer KA Felton GA Saunders R Ward DS Dowda M Pate RR Physical self-concept and self-esteem mediate cross-sectional relations of physical activity and sport participation with depression symptoms among adolescent girls Health Psychol 2006 25 3 396 407 16719612 10.1037/0278-6133.25.3.396

[B35] van Beurden E Zask A Barnett LM Dietrich UC Fundamental movement skills - how do primary school children perform? The ‘Move it Groove it’ program in rural Australia J Sci Med Sport 2002 5 3 244 252 10.1016/S1440-2440(02)80010-X 12413042

[B36] de Vet E de Ridder DT de Wit JB Environmental correlates of physical activity and dietary behaviours among young people: a systematic review of reviews Obes Rev 2011 12 5 130 142 10.1111/j.1467-789X.2010.00784.x 20630024

[B37] Van der Horst K Paw MJCA Twisk JWR Van Mechelen W A brief review on correlates of physical activity and sedentariness in youth Med Sci Sports Exerc 2007 39 8 1241 1250 10.1249/mss.0b013e318059bf35 17762356

[B38] Crawford D The future of sport in Australia 2009 Canberra: Commonwealth of Australia

[B39] Morgan PJ Hansen V Recommendations to improve primary school PE: the classroom teacher’s perspective J Educ Res 2007 101 2 99 112 10.3200/JOER.101.2.99-112

[B40] Morgan PJ Hansen V Classroom teachers’ perceptions of the impact of barriers to teaching PE on the quality of PE programs delivered in primary schools Res Q Exerc Sport 2008 79 506 516 19177952 10.1080/02701367.2008.10599517

[B41] Campbell MK Elbourne DR Altman DG CONSORT statement: extension to cluster randomised trials BMJ 2004 328 7441 702 708 10.1136/bmj.328.7441.702 15031246 PMC381234

[B42] Kriemler S Zahner L Schindler C Meyer U Hartmann T Hebestreit H Brunner-La Rocca HP van Mechelen W Puder JJ Effect of school based physical activity programme (KISS) on fitness and adiposity in primary schoolchildren: cluster randomised controlled trial BMJ 2010 340 c785 10.1136/bmj.c785 20179126 PMC2827713

[B43] Reed KE Warburton DE Macdonald HM Naylor PJ McKay HA Action Schools! BC: a school-based physical activity intervention designed to decrease cardiovascular disease risk factors in children Prev Med 2008 46 6 525 531 10.1016/j.ypmed.2008.02.020 18377970

[B44] Ulrich DA Test of Gross Motor Development Examiner’s Manual 2000 2 Austin, Texas: Pro.Ed

[B45] McLeroy KR Bibeau D Steckler A Glanz K An ecological perspective on health promotion programs Health Educ Q 1988 15 4 351 377 10.1177/109019818801500401 3068205

[B46] Deci EL Ryan RM Intrinsic motivation and self-determination in human behavior 1985 New York: Plenum Press

[B47] Deci EL Ryan RM The “what” and “why” of goal pursuits: Human needs and the self-determination of behavior Psychol Inq 2000 11 227 268 10.1207/S15327965PLI1104_01

[B48] Weiss MR Motivating kids in physical activity Pres Counc Phys Fit Sports Res Dig 2000 3 11 1 8 PMC3022443 21253445

[B49] Harter S Kolligian J, Strenberg R Competence as a dimension of self-evaluation: Toward a comprehensive model of self-worth Perceptions of competence and incompetence across the lifespan 1985 New Haven: Yale University Press

[B50] Ntoumanis N A self-determination approach to the understanding of motivation in physical education Brit J Educ Psych 2001 71 2 225 242 10.1348/000709901158497 11449934

[B51] Taylor IM Lonsdale C Cultural differences in the relationships among autonomy support, psychological need satisfaction, subjective vitality, and effort in British and Chinese physical education J Sport Exerc Psych 2010 32 5 655 673 10.1123/jsep.32.5.655 20980709

[B52] Lonsdale C Sabiston CM Raedeke TD Ha ASC Sum RKW Self-determined motivation and students’ physical activity during structured physical education lessons and free choice periods Prev Med 2009 48 69 73 10.1016/j.ypmed.2008.09.013 18996143

[B53] Hagger MS Chatzisarantis N Barkoukis V Wang CKJ Baranowski J Perceived autonomy support in physical education and leisure-time physical activity: A cross-cultural evaluation of the trans-contextual model J Educ Psych Rev 2005 97 3 376 390

[B54] Murray DM Design and analysis of group-randomized trials 1998 New York, NY: Oxford University Press

[B55] Trost SG McIvor KL Pate RR Conducting accelerometer-based activity assessments in field-based research Med Sci Sports Exerc 2005 37 suppl 11 S531 S543 16294116 10.1249/01.mss.0000185657.86065.98

[B56] Evenson KR Cattellier D Gill K Ondrak K McMurray RG Calibration of two objective measures of physical activity for children J Sports Sci 2008 26 1557 1565 18949660 10.1080/02640410802334196

[B57] Leger L Lambert JA A maximal multistage 20 m shuttle run test to predict VO2 max Eur J Appl Physiol 1982 49 1 12 10.1007/BF00428958 7201922

[B58] Cole TJ Bellizzi MC Flegal KM Dietz WH Establishing a standard definition for child overweight and obesity worldwide: international survey BMJ 2000 320 7244 1240 10.1136/bmj.320.7244.1240 10797032 PMC27365

[B59] Harter S Manual for the self-perception profile for children 1985 University of Denver, Denver

[B60] Resilience Research Centre The Child and Youth Resilience Measure-28: User Manual 2009 Halifax, NS: Resilience Research Centre, Dalhousie University

[B61] Ungar M Liebenberg L Assessing resilience across cultures using mixed-methods: construction of the Child and Youth Resilience Measure-28 J Mix Methods Res 2011 5 2 126 149 10.1177/1558689811400607

[B62] Roberts C Currie C Samdal O Currie D Smith R Maes L Measuring the health and health behaviours of adolescents through cross-national survey research: recent developments in the Health Behaviour in School-aged Children (HBSC) study J Pub Health 2007 15 179 186 10.1007/s10389-007-0100-x

[B63] Lubans DR Hesketh K Cliff DP Barnett LM Salmon J Dollman J Morgan PJ Hills AP Hardy LL A systematic review of the validity and reliability of sedentary behaviour measures used with children and adolescents Obes Rev 2011 12 10 781 799 10.1111/j.1467-789X.2011.00896.x 21676153

[B64] Liu Y Wang M Tynjälä J Lv Y Villberg J Zhang Z Kannas L Test-retest reliability of selected items of Health Behaviour in School-aged Children (HBSC) survey questionnaire in Beijing, China BMC Med Res Methodol 2010 10 73 10.1186/1479-5868-1187-1137 PMC2927607 20696078

[B65] Lubans DR Foster C Biddle SJH A review of mediators of behavior in interventions to promote physical activity among children and adolescents Prev Med 2008 47 463 470 10.1016/j.ypmed.2008.07.011 18708086

[B66] Rhodes RE Pfaeffli LA Mediators of physical activity behaviour change among adult non-clinical populations: a review update Int J Behav Nutr Phys Act 2010 7 37 10.1186/1479-5868-1187-1137 PMC2876989 20459781

[B67] Motl RW Dishman RK Saunders RP Dowda M Felton G Pate RR Measuring enjoyment of physical activity in adolescent girls Am J Prev Med 2001 21 2 110 117 10.1016/S0749-3797(01)00326-9 11457630

[B68] Prochaska JJ Rodgers MW Sallis JF Association of parent and peer support with adolescent physical activity Res Q Exerc Sport 2002 73 2 206 210 12092896 10.1080/02701367.2002.10609010

[B69] Lubans DR Morgan PJ McCormack A Adolescents and school sport: The relationship between beliefs, social support and physical self-perception Phys Educ Sport Pedagogy 2011 16 3 237 250 10.1080/17408989.2010.532784

[B70] Telford A Salmon J Jolley D Crawford D Reliability and validity of physical activity questionnaires for children: The Children’s Leisure Activities Study Survey (CLASS) Pediatr Exerc Sci 2004 16 1 64 78 10.1123/pes.21.3.339 19827457

[B71] Hardy L King L Espinel P Okely AD Baumanb A Methods of the NSW SchoolsPhysical Activity and Nutrition Survey 2010 (SPANS 2010) J Sci Med Sport 2011 14 5 390 396 10.1016/j.jsams.2011.03.003 21454126

[B72] McKenzie TL Sallis JF Nader PR SOFIT: System for Observing Fitness Instruction Time J Teach Phys Educ 1991 11 195 205

[B73] Krull JL MacKinnon DP Multilevel modeling of individual and group level mediated effects Multivariate Behav Res 2001 36 249 277 10.1207/S15327906MBR3602_06 26822111

[B74] Sallis JF Age-related decline in physical activity: A synthesis of human and animal studies Med Sci Sport Exerc 2000 32 9 1598 1600 10.1097/00005768-200009000-00012 10994911

[B75] Resaland GK Anderssen SA Holme IM Mamen A Andersen LB Effects of a 2-year school-based daily physical activity intervention on cardiovascular disease risk factors: the Sogndal school-intervention study Scand J Med Sci Sports 2011 21 6 122 131 10.1111/j.1600-0838.2010.01181.x 22126720

[B76] Morgan PJ Hansen V Physical education in primary schools: Classroom teachers’ perceptions of benefits and outcomes Health Educ J 2008 67 196 207 10.1177/0017896908094637

[B77] Avalos B Teacher professional development in Teaching and Teacher Education over ten years Teaching Teach Educ 2011 27 10 20 10.1016/j.tate.2010.08.007

